# Editorial: Perinatal assessment of biomarkers in invasive and non-invasive procedures of biological fluid collection

**DOI:** 10.3389/fped.2022.1010205

**Published:** 2022-09-06

**Authors:** Iliana Bersani, Diego Gazzolo, Fiammetta Piersigilli

**Affiliations:** ^1^Department of Medical and Surgical Neonatology, Bambino Gesù Children's Hospital, Rome, Italy; ^2^Neonatal Intensive Care Unit, G. d'Annunzio University, Chieti, Italy; ^3^Section of Neonatology, Cliniques Universitaires Saint Luc, Université Catholique de Louvain, Bruxelles, Belgium

**Keywords:** neonate, biomarker, biological fluids, theranostics, diagnosis

During the last decades, the number of studies focusing on the investigation and validation of biomarkers increased exponentially. To date, however, significant confusion persists about the concept of “biomarkers” and their appropriate validation, qualification and application (1–3). As established by the U.S. Food and Drug Administration and the National Institutes of Health as part of their joint Biomarkers, EndpointS, and other Tools (BEST) resource, a biomarker is “a defined characteristic that is measured as an indicator of normal biological processes, pathogenic processes or responses to an exposure or intervention” (4). The underlying concept of the BEST approach is that the appropriate use of biomarkers could allow higher precision, speed and efficacy in the diagnostic/therapeutic strategies (2, 5). European Medicines Agency (6) defines a biomarker as a “biological molecule found in blood, other body fluids, or tissues that can be used to follow body processes and diseases in humans and animals” (6). Biomarkers are considered to be “surrogate endpoints”, meaning that they act as surrogates or substitutes for clinically relevant endpoints (1, 3). However, only a minority of biomarkers can be actually considered a surrogate endpoint, since a solid and relevant evidence demonstrating that a biomarker accurately predicts a clinical outcome is required (1). For this reason, researches having the aim to assess the role of biomarkers as suitable diagnostics/therapeutic tools are of crucial importance.

In the last years, the international health-care institutions promoted clinical and laboratory researches focused on the use of biomarkers in the perinatal period. Perinatologists and researchers have therefore the objective to identify perinatal biomarkers ranges and to describe their behavior from first detection of a disease at earlier stages toward disease progression through time, with the final aim of developing suitable interventions to ameliorate patient care and treatment. In fact, the clinical qualification and validation of biomarkers would allow to identify precociously newborns at higher risk for a specific disease, allow a stratification of patients according to disease severity, enhance the use of personalized preventive or therapeutic treatment strategies, and guide caregivers in daily practice and clinical decision-making path (7, 8). The final aim being the development of theranostics, that tailors optimized therapies based on the patient's biomarkers profile, improving response to treatment and reducing side effects and finally increasing the quality of clinical care by identifying the right drug for the right patient at the right time.

The optimal biomarker should be easily collectable, reproducible, able to predict precociously injury degree, extent and location, or able to monitor longitudinally disease progression and to correlate with standard procedures such as imaging techniques. Concerning the latter, the optimal biomarker should possibly represent an alternative and direct indicator of damage when clinical and radiologic assessments are still silent (9). The ideal biomarker should possibly be identifiable in different biologic fluids (amniotic fluid, cerebrospinal fluid, blood, urine, bronchoalveolar lavage fluid, saliva, milk) and its assessment should be obtained worldwide and with good reproducibility by available commercial kits. Furthermore, due to the complexity of the pathogenesis of certain neonatal diseases (BPD, NEC, ROP), the use of only one biomarker could be insufficient, but it would be rather necessary to develop a panel of biomarkers to allow identify those neonates that are prone to develop a specific disease (10).

To achieve a proper utilization of the biomarker, its assessment should be relatively low-cost and widely available (9) and to avoid inappropriate interpretation of biomarker measurements, the availability of reference curves specific for the perinatal period should be considered, and the ranges of normality of the ideal biomarker should be available both for healthy term and preterm neonates (11). Such age-specific reference values are of crucial importance considering that the neonatal period represents a unique life period with peculiar physiologic and biologic features which reflect the transitional and developing aspects of the organism.

In the neonatal setting, possible limitations to the use of biomarkers may include the small study cohort sizes, the heterogeneity of the population, the lack of specific reference curves for the neonatal period, the sample volume required, the time for results to be ready, the lack of stratification according to disease severity. Moreover, the sampling costs might be an obstacle for a wide use of biomarkers in the perinatal period, although the costs/benefits of using biochemical markers in daily practice may be lower than those of any of the standard procedures used for disease monitoring in neonate and would support a proper selection of candidates (3).

Considering that scientific evidence needs to be in place before a biomarker is used routinely in clinical practice, an implementation of precision medicine based on the use of perinatal biomarkers (11) is at the forefront for a targeted and personalized approach to perinatal disease, and deserves maximal attention from the scientific community.

## Author contributions

All authors listed have made a substantial, direct, and intellectual contribution to the work and approved it for publication.

## Conflict of interest

The authors declare that the research was conducted in the absence of any commercial or financial relationships that could be construed as a potential conflict of interest.

## Publisher's note

All claims expressed in this article are solely those of the authors and do not necessarily represent those of their affiliated organizations, or those of the publisher, the editors and the reviewers. Any product that may be evaluated in this article, or claim that may be made by its manufacturer, is not guaranteed or endorsed by the publisher.
